# Supplementary material for: Polyketide synthase gene clusters in the ericoid mycorrhizal fungus Oidiodendron maius: genome-wide prediction, expression profiling, and functional characterization of knockout mutants
Source: Front Plant Sci. 2026 Sep 16;17:1924430. doi: 10.3389/fpls.2026.1924430 (PMC13623903; doi:10.3389/fpls.2026.1924430)
Supplement: Supplementary file 1 [file SupplementaryFile1.zip › Supplementary Materials/Supplementary Folder 1.zip!/Oidma1_AssemblyScaffolds_Repeatmasked/knownclusterblast/cluster21/jgi.p_Oidma1_42470_mibig_hits.html]

| MIBiG Protein | Description | MIBiG Cluster | MiBiG Product | % ID | % Coverage | BLAST Score | E-value |
| --- | --- | --- | --- | --- | --- | --- | --- |
| CAP95407.1 |  | BGC0001404 | Sorbicillin\_biosynthetic\_gene\_cluster | 64 | 95.4455445545 | 635 | 4.5e-182 |
| EWG54280.1 | hypothetical\_protein | BGC0001190 | Fusaric\_acid\_biosynthetic\_gene\_cluster | 52 | 94.8514851485 | 504 | 1.2e-142 |
| KFH48706.1 | putative\_MFS-type\_transporter-like\_protein | BGC0000317 | Cephalosporin\_biosynthetic\_gene\_cluster | 41 | 99.0099009901 | 364 | 1.9e-100 |
| EED18004.1 | conserved\_hypothetical\_protein | BGC0000154 | Stipitatic\_acid\_biosynthetic\_gene\_cluster | 37 | 91.4851485149 | 314 | 1.8e-85 |
| ACD39772.1 | major\_facilitator\_superfamily\_transporter | BGC0000134 | Radicicol\_biosynthetic\_gene\_cluster | 36 | 90.495049505 | 286 | 5.2e-77 |
| EAU32817.1 | conserved\_hypothetical\_protein | BGC0000160 | Terreic\_acid\_biosynthetic\_gene\_cluster | 35 | 84.7524752475 | 279 | 6.3e-75 |
| BAK26557.1 | putative\_MFS\_multidrug\_transporter | BGC0000977 | Cyclopiazonic\_acid\_biosynthetic\_gene\_cluster | 35 | 90.495049505 | 279 | 1.1e-74 |
| ABK64181.1 | MFS\_transporter | BGC0001247\_c4 | Cercosporin\_biosynthetic\_gene\_cluster | 34 | 96.0396039604 | 277 | 4.1e-74 |
| KFH48645.1 | putative\_transporter\_-like\_protein | BGC0000317 | Cephalosporin\_biosynthetic\_gene\_cluster | 35 | 91.6831683168 | 275 | 1.2e-73 |
| CCT67996.1 | bikaverin\_cluster-efflux\_pump | BGC0000030 | Bikaverin\_biosynthetic\_gene\_cluster | 36 | 91.8811881188 | 275 | 1.2e-73 |
| CCH26293.1 | putative\_MFS-type\_transporter | BGC0001158\_c4 | Bikaverin\_biosynthetic\_gene\_cluster | 36 | 91.8811881188 | 273 | 4.5e-73 |
| EAQ86381.1 | hypothetical\_protein | BGC0001405 | Chaetoviridin\_/\_chaetomugilin\_biosynthetic\_gene\_cluster | 33 | 90.099009901 | 260 | 3e-69 |
| AGO86666.1 | putative\_MFS\_transporter | BGC0001255 | Equisetin\_biosynthetic\_gene\_cluster | 35 | 91.4851485149 | 250 | 3.1e-66 |
| AGO65989.1 | putative\_MFS\_transporter | BGC0000992 | Fusaridione\_A\_biosynthetic\_gene\_cluster | 33 | 91.6831683168 | 239 | 7.2e-63 |
